# Associations of magnesium intake and a magnesium-rich diet score with sarcopenia in middle-aged and older Korean adults: potential mediation by C-reactive protein

**DOI:** 10.3389/fnut.2026.1854192

**Published:** 2026-08-03

**Authors:** Sooyeun Choi, Youjin Je

**Affiliations:** Department of Food and Nutrition, Kyung Hee University, Seoul, Republic of Korea

**Keywords:** chronic inflammation, C-reactive protein, magnesium, magnesium-rich diet score, sarcopenia

## Abstract

**Background/objectives:**

Dietary magnesium, primarily derived from plant foods, has been inconsistently associated with sarcopenia, and the underlying mechanisms have rarely been explored. Although foods and dietary patterns may act synergistically, to our knowledge, no previous study has jointly examined magnesium intake and a magnesium-rich dietary pattern in relation to sarcopenia. We investigated whether dietary magnesium intake and a magnesium-rich diet score were associated with sarcopenia in middle-aged and older Korean adults and whether chronic inflammation might partially mediate these associations.

**Methods:**

A cross-sectional analysis was conducted using data from the Korea National Health and Nutrition Examination Survey 2022–2024, including 3,830 adults aged ≥50 years. Dietary intake was assessed using a 24-h dietary recall. A magnesium-rich diet score was calculated based on the intake of whole grains, nuts and legumes, green leafy vegetables and seaweeds, soymilk, and coffee.

**Results:**

After full adjustment for covariates, including diet quality, the highest quartile of magnesium intake (≥351 mg/day for women and ≥442 mg/day for men) was inversely associated with sarcopenia [odds ratio (OR) = 0.47, 95% confidence interval (CI): 0.23–0.95], compared with the lowest quartile. The highest quartile of the magnesium-rich diet score was also inversely associated with sarcopenia (OR = 0.50, 95% CI: 0.30–0.84), compared with the lowest quartile. The inverse associations were consistently observed for each 100 mg/day increase in magnesium intake and for each 1-point increase in the magnesium-rich diet score. High-sensitivity C-reactive protein (hsCRP), a marker of chronic inflammation, appeared to partially mediate the association between magnesium intake and greater muscle mass, with a proportion mediated of 7.3%. hsCRP also appeared to partially mediate the associations of the magnesium-rich diet score with greater muscle mass and muscle strength, with proportions mediated of 18.1 and 8.6%, respectively (all *p* < 0.05).

**Conclusion:**

Our findings suggest that higher magnesium intake and a higher magnesium-rich diet score are associated with lower odds of sarcopenia among middle-aged and older Korean adults and that lower chronic inflammation may partially mediate these associations.

## 1 Introduction

Given that the global proportion of the population aged ≥60 years is predicted to approximately double from 12 to 22% between 2015 and 2050 (1), prevention and treatment strategies for geriatric diseases, including sarcopenia, are increasingly required. The Asian Working Group for Sarcopenia (AWGS) 2025 Consensus Update reported that sarcopenia is defined as age-related loss of skeletal muscle mass plus low muscle strength (2). Sarcopenia is associated with fall-related injuries, osteoporosis, cardiovascular disease, cognitive impairment, prolonged rehabilitation, and all-cause mortality (3–7). The prevalence of sarcopenia ranges from 8 to 27% among individuals aged ≥60 years in the studies included in a previous meta-analysis (8). In Korea, in 2022, the prevalence of low handgrip strength among individuals aged ≥65 years was 14.2% in men and 18.8% in women, while the prevalence of sarcopenia was 6.6% in men and 9.2% in women (9).

Sarcopenia was promulgated as a disease state under the International Classification of Diseases, Clinical Modification (ICD-10-CM) code M62.84 in 2016 (10). To date, no pharmaceutical interventions have been approved for the treatment of sarcopenia (2). The AWGS 2025 recommends multimodal interventions focusing on resistance exercise and protein supplementation or a protein-rich diet for the management of sarcopenia, while noting that findings from studies on vitamin D and muscle health remain equivocal. The updated European Working Group on Sarcopenia in Older People (EWGSOP2) recommends optimal protein intake, vitamin D supplementation, and physical exercise as treatment options for sarcopenia (11). Neither guideline addresses minerals, possibly because the cumulative evidence has largely focused on protein and vitamin D (2).

Magnesium is primarily derived from plant-based sources (e.g., green leafy vegetables, unrefined grains, nuts and legumes, coffee, and soymilk) (12, 13). Magnesium plays an important role in myogenesis via the activation of the mammalian target of rapamycin (mTOR), muscle contraction, and muscle performance. It also forms a complex that biologically activates ATP (14, 15). Magnesium also modulates chronic inflammation, which is a risk factor for sarcopenia (15, 16). Because magnesium is mainly stored in bones (50–60%) and muscles (approximately 25%), circulating magnesium concentrations may not accurately reflect total body magnesium levels, leading to the frequent underdetection of magnesium deficiency (14).

The findings of several studies examining the association between magnesium intake and sarcopenia (17) or muscle-specific parameters (18–21) are inconsistent, with some reporting a protective association (17–19) and others reporting no significant association (20, 21). To our knowledge, no study has examined the potential mediating role of chronic inflammation in this association; only one study suggested that the inverse association between high-sensitivity C-reactive protein (hsCRP) concentrations and skeletal muscle mass was modestly attenuated after additional adjustment for magnesium intake (18). C-reactive protein (CRP) is an acute-phase protein synthesized by hepatocytes in response to pro-inflammatory cytokines and serves as a useful marker for evaluating chronic inflammation (22). Meta-analyses and large-scale studies have reported that dietary magnesium intake or magnesium supplementation is inversely associated with CRP levels (23–25), while elevated CRP levels are associated with lower skeletal muscle strength and muscle mass (26, 27). In addition, Korean research on magnesium intake and sarcopenia is limited to a single descriptive study in older adults (28). Given that cumulative evidence suggests that foods and dietary patterns may act synergistically to influence the risk of several chronic diseases, a magnesium-rich dietary pattern may better predict disease risk than magnesium intake alone (29). Individuals consume meals composed of multiple foods containing various nutrients, which are likely to interact synergistically. Nutrient-focused research may contribute to elucidating the mechanisms underlying diet effects. Taken together, evaluating both magnesium intake and a magnesium-rich dietary pattern in relation to sarcopenia risk appears to be a complementary approach (29, 30). A previous study reported that a magnesium-rich diet score was associated with lower risks of cardiovascular disease and coronary heart disease, whereas dietary magnesium intake was not associated with these outcomes (13). To our knowledge, no previous study has jointly examined magnesium intake and a magnesium-rich diet score in relation to sarcopenia.

Therefore, we aimed to evaluate the associations of dietary magnesium intake and a magnesium-rich diet score with sarcopenia in middle-aged and older Korean adults and to examine the potential role of chronic inflammation in these associations, using data from the Korea National Health and Nutrition Examination Survey (KNHANES) and the updated AWGS diagnostic criteria for sarcopenia.

## 2 Materials and methods

### 2.1 Study population

**Figure 1:**
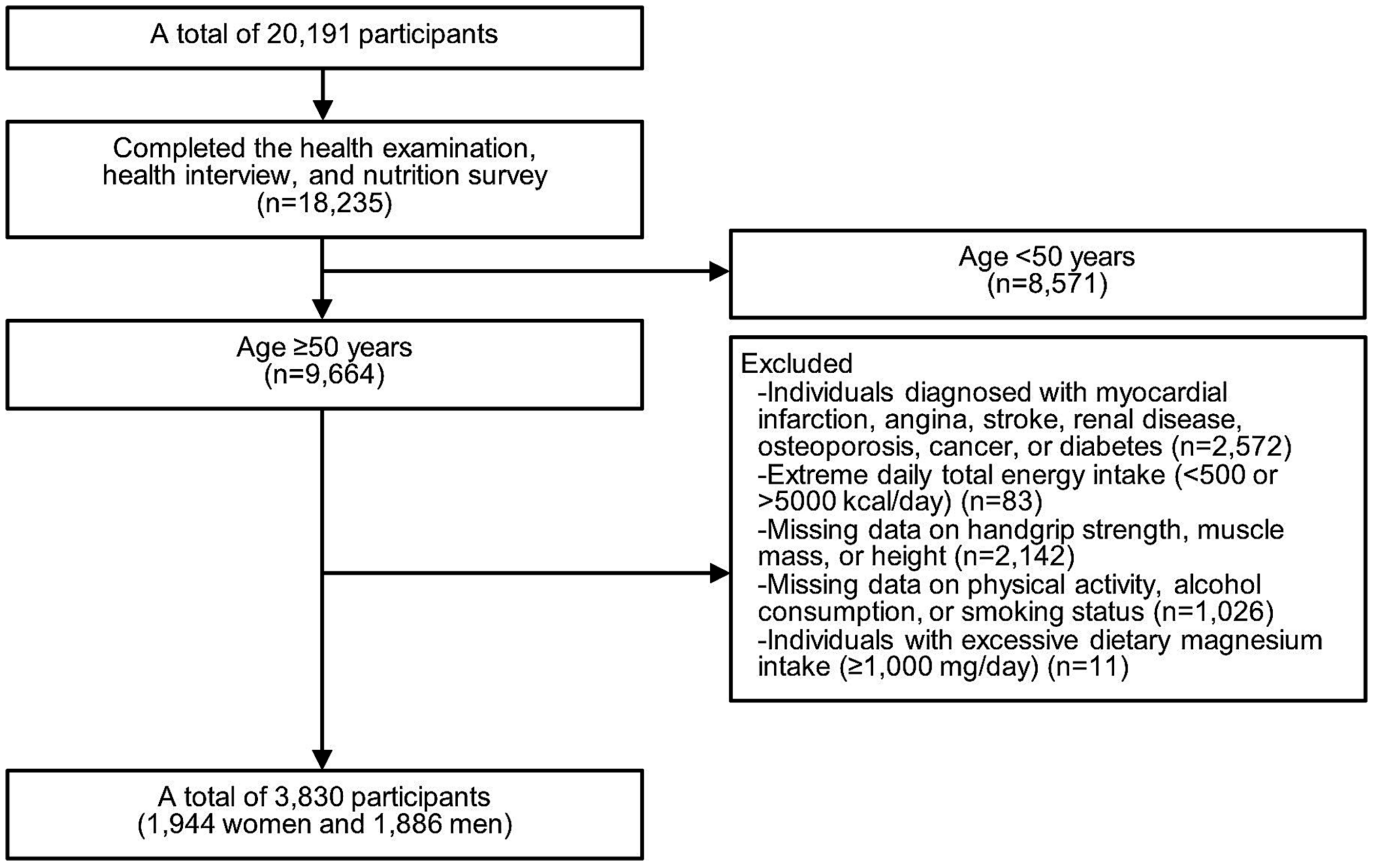
Study population aged ≥50 years after applying the exclusion criteria.

This study utilized data from the ninth period (2022–2024) of the KNHANES, a nationally representative cross-sectional survey conducted by the Korea Disease Control and Prevention Agency (KDCA). The KNHANES adopts a multistage, clustered probability sampling design and includes non-institutionalized Korean civilians. Data were collected from 20,191 participants during the ninth period of the KNHANES. Among the participants, we included 18,235 individuals who completed all three components of the KNHANES: the health examination, health interview, and nutrition survey. We then sequentially excluded the following participants: 8,571 individuals aged <50 years; 2,572 individuals who reported a diagnosis of myocardial infarction, angina, stroke, renal disease, osteoporosis, cancer, or diabetes; 83 individuals with extreme daily total energy intake (<500 or >5,000 kcal/day); 2,142 individuals with missing data on handgrip strength, appendicular skeletal muscle mass (ASM), or height; 1,026 individuals with missing data on major confounders, including physical activity, alcohol consumption, or smoking status; and 11 individuals with excessive magnesium intake (≥1,000 mg/day). Based on the AWGS 2025 Consensus, which provides age-specific cutoffs for individuals aged 50–64 years and ≥65 years, we restricted the study population to individuals aged ≥50 years (2). Finally, we included 3,830 participants, consisting of 1,944 women and 1,886 men. A flow diagram of the study population is presented in Figure 1. The KDCA obtained informed consent from all participants, and the Institutional Review Board of the KDCA approved the KNHANES datasets (2018-01-03-4C-A, 2022-11-16-R-A, and 2022-11-16-R-03).

### 2.2 Assessment of dietary magnesium intake and magnesium-rich diet score

The 24-h dietary recall data from KNHANES were used to assess dietary intake. Estimates of magnesium intake from supplements remain limited in KNHANES, possibly because the prevalence of magnesium supplement use is relatively low (31). Moreover, specific supplement use was not surveyed in KNHANES 2022–2024. We categorized dietary magnesium intake into sex-specific quartiles based on the distribution of magnesium intake in our study population and the methods used in previous studies examining magnesium intake in relation to muscle-related outcomes (32, 33).

The magnesium-rich food groups were defined based on prior research and official guidance on dietary sources of magnesium as follows (12, 13): (1) whole grains; (2) nuts, legumes, and seeds; (3) green leafy vegetables and seaweeds; (4) soymilk; and (5) coffee. To reduce the risk of misinterpretation when summing intakes across foods in different physical states, we used food intake data for the tertiary food codes, calculated using solid-content-based conversion coefficients. The magnesium-rich diet score was calculated by summing scores assigned according to quintiles of intake for each magnesium-rich food group (quintile 1 = 1, quintile 2 = 2, quintile 3 = 3, quintile 4 = 4, and quintile 5 = 5). The component scores of the magnesium-rich diet score were weighted equally, consistent with previous studies using a magnesium-rich diet score (13) or other commonly used dietary pattern indices, such as plant-based diet indices and Mediterranean diet scores (34–36). The total magnesium-rich diet score theoretically ranged from 5 to 25 and was then categorized into quartiles for further statistical analyses (13).

### 2.3 Assessment of sarcopenia and muscle-specific parameters

Handgrip strength (kg) was measured twice in the KNHANES using a digital grip strength dynamometer (T. K. K. 5,401, Japan). Participants who were screened by visual inspection for defects of the arm, hand, or thumb, or by questionnaire for a history of hand or wrist surgery within the previous 3 months, did not undergo the handgrip strength test. Body composition, including muscle mass (kg), was measured using a bioelectrical impedance analyzer (InBody 970, Biospace, Korea). Individuals with implanted cardiac devices, such as pacemakers or implantable cardioverter-defibrillators, and pregnant women did not undergo the body composition test. Height (cm) was measured using a stadiometer (Seca 274, Germany) and recorded to the nearest 0.1 cm. Participants stood barefoot with their heels, buttocks, shoulders, and the back of the head in contact with the vertical board. To obtain height-adjusted ASM (kg/m^2^), we divided ASM (kg), defined as the sum of the muscle mass of the left and right arms and legs, by height squared (m^2^). In this study, the maximum handgrip strength value (kg) and height-adjusted ASM (kg/m^2^) were used for the statistical analysis (2).

According to the AWGS 2025 Consensus, for individuals aged ≥65 years, sarcopenia was defined by meeting both of the following diagnostic criteria: (1) handgrip strength <18 kg for women and <28 kg for men; and (2) height-adjusted ASM < 5.7 kg/m^2^ for women and <7.0 kg/m^2^ for men. For individuals aged 50–64 years, sarcopenia was defined by meeting both of the following diagnostic criteria: (1) handgrip strength <20 kg for women and <34 kg for men; and (2) height-adjusted ASM < 5.7 kg/m^2^ for women and <7.6 kg/m^2^ for men. In this study, low muscle strength only was defined as meeting the criterion for low handgrip strength while not meeting the criterion for low height-adjusted ASM. Low muscle mass only was defined as meeting the criterion for low height-adjusted ASM while not meeting the criterion for low handgrip strength.

### 2.4 Covariates and other variables

Demographic, lifestyle, and health-related variables were assessed through interviews, self-reported questionnaires, or examinations. Physical activity was categorized as low or high. High physical activity was defined as ≥150 min per week of moderate-intensity activity, ≥75 min per week of vigorous-intensity activity, or ≥150 min per week of a combination of moderate and vigorous-intensity activity. One minute of vigorous activity was regarded as equivalent to 2 minutes of moderate activity. Marital status was categorized as unmarried or married. Education level was categorized into middle school or lower, high school, and college or higher. Occupation was classified as white-collar, blue-collar, or non-employed. Household income was classified into quartiles. Sleep duration was categorized into <6, 6–<8, and ≥8 h per day. Alcohol consumption was calculated by multiplying the amount of alcohol intake per occasion by the frequency of alcohol intake in the past year and was then classified as <1, 1–<3, and ≥3 drinks per day. Smoking status was categorized as non-smoker, past smoker, and current smoker. Diet quality was assessed using a modified Diet Quality Index for Koreans (DQI-K) and classified as poor or good. DQI-K points, ranging from 0 to 1 or 2, were assigned to each of the eight dietary factors according to the 2025 Dietary Reference Intakes for Koreans (KDRIs) (37, 38). The total DQI-K score, defined as the sum of all factor points, ranged from 0 to 9, with scores of 0–4 reflecting good diet quality and scores of 5–9 reflecting poor diet quality. Daily protein intake (g/day) and daily total energy intake (kcal/day) were assessed using 24-h dietary recall data. Estimated glomerular filtration rate (eGFR) was calculated using the 2021 CKD-EPI creatinine–age–sex equation. Serum hsCRP levels (mg/L) were measured using a particle-enhanced immunoturbidimetric assay on the Cobas 8,000 analyzer with the Cardiac C-Reactive Protein (Latex) High Sensitive reagent (Roche, Germany). Serum vitamin D levels (ng/mL) were calculated as the sum of 25-hydroxyvitamin D₂ and 25-hydroxyvitamin D₃, measured using liquid chromatography–tandem mass spectrometry on a Triple Quad 4,500 MD system (SCIEX, United States) with the MSMS Vitamin D kit (PerkinElmer, Finland). In the KNHANES, when 25-hydroxyvitamin D₂ levels were below 0.30 ng/mL, the continuous variable HE_D2 was assigned a missing value, and the categorical variable HE_D2_etc was assigned “<0.30.” In such cases, we replaced the missing value with 0.29 in the continuous variable HE_D2 to ensure data consistency.

### 2.5 Statistical analysis

For categorical variables, numbers and weighted percentages were obtained. For continuous variables, means with standard errors (SEs) were calculated. The chi-square test was used to compare categorical variables across magnesium intake or the magnesium-rich diet score, and analysis of variance (*F*-test) was used to compare continuous variables across magnesium intake or the magnesium-rich diet score. To obtain multivariable-adjusted odds ratios (ORs) and 95% confidence intervals (CIs) for sarcopenia, low muscle strength only, and low muscle mass only according to magnesium intake and the magnesium-rich diet score, we used multinomial logistic regression models with the PROC SURVEYLOGISTIC procedure. All descriptive analyses and logistic regression models accounted for the complex survey design of KNHANES by applying the appropriate strata, primary sampling units, and sample weights. To examine the potential mediating role of hsCRP in the associations of magnesium intake and the magnesium-rich diet score with muscle-specific parameters, including handgrip strength (kg) and height-adjusted ASM (kg/m^2^), we conducted mediation analyses with the PROC CAUSALMED procedure. A natural log transformation was applied to hsCRP to better approximate a normal distribution. The mediation analyses decomposed the total effect of each continuous exposure—magnesium intake (per 100 mg/day increase) and the magnesium-rich diet score (per 1-point increase)—on each continuous outcome—handgrip strength (kg) and height-adjusted ASM (kg/m^2^)—into natural direct and natural indirect effects. The natural indirect effect reflects the portion of the association that is mediated by the studied marker (hsCRP in this study), and the natural direct effect reflects the portion that operates directly or through pathways other than the studied marker. Given the observational nature of this study, the term “effect,” as used in mediation analyses, should not be interpreted as a causal effect. Results of the mediation analyses are presented as effect estimates (*β*) with corresponding 95% CIs and as the proportion mediated (%).

Model 1 was adjusted for age (continuous) and sex (women and men). Model 2 was adjusted for age, sex, body mass index (BMI; kg/m^2^, continuous), eGFR (mL/min/1.73 m^2^, continuous), physical activity (low or high), marital status (unmarried or married), education level (middle school or lower, high school, and college or higher), occupation (white-collar, blue-collar, or non-employed), household income (lowest, lower-middle, upper-middle, and highest quartiles), sleep duration (<6, 6–<8, and ≥8 h/day), alcohol consumption (<1, 1–<3, and ≥3 drinks/day), smoking status (non-smoker, past smoker, and current smoker), DQI-K (poor or good diet quality), daily protein intake (g/day, continuous), and daily total energy intake (kcal/day, continuous). When selecting covariates, we considered previous research related to this topic and the characteristics of our study population (17–20, 39–41). Potential effect modification by sex and vitamin D status normal (≥20 ng/mL) or deficient (< 20 ng/mL), (42) was tested. A recent study involving older adults undergoing geriatric rehabilitation in Switzerland found a positive association between serum magnesium levels and handgrip strength only among vitamin D–sufficient individuals (43). A cross-product term between magnesium intake quartiles and each potential modifier was added to the fully adjusted model. Because we did not identify significant interactions with sex or vitamin D status, we did not conduct further stratified analyses by these variables.

We conducted additional analyses to examine the associations of protein intake and serum vitamin D levels with sarcopenia, low muscle mass only, and low muscle strength only. These nutrients are commonly discussed as potential nutritional intervention approaches in recent guidelines (2, 11). We also explored whether the association between magnesium intake and sarcopenia was independent of serum vitamin D levels, with further adjustment for serum vitamin D levels; daily protein intake was already included as a covariate in the main models. All statistical analyses were performed using SAS software (version 9.4; SAS Institute Inc., Cary, NC, United States). A two-tailed *p*-value of < 0.05 was considered statistically significant.

## 3 Results

### 3.1 General characteristics

Table 1 presents the general characteristics of middle-aged and older Korean adults according to magnesium intake. The mean age of participants was 62.95 years [standard error (SE): 0.23]. Compared with individuals in the lowest quartile, those in the highest quartile of magnesium intake were more likely to have higher household income and shorter sleep duration. Individuals with higher magnesium intake had higher serum vitamin D levels, daily protein intake, and daily total energy intake. Those with higher magnesium intake were less likely to be current smokers and more likely to have higher education levels, physical activity, and diet quality.

**Table 1:** Characteristics of the study population according to sex-specific quartiles of magnesium intake among middle-aged and older Korean adults aged ≥50 years^a^.

Magnesium intake									
	Q1 (Lowest)		Q2		Q3		Q4 (Highest)		*P* ^b^
Characteristics	Mean or *n*	SE or %	Mean or *n*	SE or %	Mean or *n*	SE or %	Mean or *n*	SE or %	
*n*	957		958		958		957		
Age (years)	63.15	0.40	62.48	0.36	62.67	0.34	63.51	0.35	0.104
Sex (%)									0.947
Men	471	53.32	472	53.21	472	52.87	471	51.90	
Women	486	46.68	486	46.79	486	47.13	486	48.10	
Magnesium intake (mg/day)									
Total	185	1.79	273	1.47	352	1.79	512	3.91	<0.001^**^
Men	210	2.49	307	1.12	391	1.35	562	5.22	<0.001^**^
Women	156	1.65	235	0.99	307	1.15	457	4.46	<0.001^**^
BMI (kg/m^2^)	23.95	0.12	24.21	0.11	24.10	0.12	24.10	0.13	0.463
eGFR (mL/min/1.73 m^2^)	91.13	0.49	92.36	0.49	92.48	0.46	92.54	0.43	0.089
Serum vitamin D levels (ng/mL)	25.61	0.49	27.29	0.46	27.26	0.39	27.38	0.45	0.016^*^
Physical activity^c^ (%)									0.001^**^
Low	610	62.66	573	57.29	579	58.24	525	52.30	
High	347	37.34	385	42.71	379	41.76	432	47.70	
Marital status (%)									0.980
Married	932	96.93	936	97.31	936	97.00	928	97.06	
Unmarried	25	3.07	22	2.69	22	3.00	29	2.94	
Occupation (%)									0.263
White-collar	269	31.7871	297	33.6446	308	35.8985	263	30.9846	
Blue-collar	266	28.2685	286	29.4196	264	26.7904	267	26.9051	
Non-employed	420	39.9445	374	36.9358	385	37.3111	427	42.1104	
Education level (%)									0.003^**^
Middle school or lower	426	38.04	362	32.31	327	28.73	338	30.63	
High school	313	35.14	337	37.37	336	36.50	324	35.94	
College or higher	217	26.82	259	30.32	295	34.77	294	33.43	
Household income (%)									<0.001^**^
Lowest	284	25.50	197	17.46	191	16.78	186	16.81	
Lower-middle	244	24.08	265	24.93	247	24.91	253	23.97	
Upper-middle	208	24.14	212	23.20	241	26.52	253	27.02	
Highest	220	26.29	283	34.41	277	31.79	263	32.20	
Sleep duration (%)									0.018^*^
<6 h/day	174	18.46	155	15.68	130	12.98	140	14.09	
6–<8 h/day	504	53.43	525	55.29	584	61.69	545	58.13	
≥8 h/day	278	28.11	277	29.04	243	25.33	270	27.79	
Alcohol consumption (%)									0.611
<1 drink/day	774	78.41	762	77.85	775	79.76	783	80.27	
1–<3 drinks/day	109	14.72	126	13.36	119	12.15	114	12.34	
≥3 drinks/day	65	6.88	57	8.80	77	8.09	69	7.39	
Smoking status (%)									<0.001^**^
Non-smoker	532	52.25	539	53.48	537	51.80	536	54.99	
Past smoker	262	29.42	271	29.14	289	33.51	324	35.07	
Current smoker	163	18.33	148	17.38	132	14.69	97	9.94	
Diet quality^d^ (%)									<0.001^**^
Poor	696	70.58	632	63.82	520	52.18	414	42.96	
Good	261	29.42	326	36.18	438	47.82	543	57.04	
Daily protein intake (g/day)	44.11	0.66	59.30	0.79	73.79	0.90	95.80	1.28	<0.001^**^
Daily total energy intake (kcal/day)	1282.19	16.68	1652.83	19.84	1942.59	22.05	2380.66	27.16	<0.001^**^

^a^Values are presented as means with standard errors (SEs) for continuous variables and as numbers with weighted percentages for categorical variables.

^b^The chi-square test was used to compare categorical variables across magnesium intake, and analysis of variance (*F*-test) was used to compare continuous variables across magnesium intake.

^c^High physical activity was defined as ≥150 min per week of moderate-intensity activity, ≥75 min per week of vigorous-intensity activity, or ≥150 min per week of a combination of moderate and vigorous-intensity activity, with 1 min of vigorous-intensity activity regarded as equivalent to 2 min of moderate-intensity activity.

^d^Diet quality was evaluated using a modified Diet Quality Index for Koreans and classified as poor (scores of 5–9) or good (scores of 0–4).

^*^p < 0.05; ^**^p < 0.01.

Table 2 presents the general characteristics of middle-aged and older Korean adults according to the magnesium-rich diet score. The mean magnesium-rich diet scores for Q1 to Q4 were 9.38 (SE: 0.02), 11.53 (SE: 0.02), 13.42 (SE: 0.02), and 15.78 (SE: 0.04), respectively. Compared with individuals in the lowest quartile, those in the highest quartile of the magnesium-rich diet score were more likely to be men, to have higher education levels, and to have shorter sleep duration. Individuals with higher magnesium-rich diet scores were older and had higher serum vitamin D levels, daily protein intake, and daily total energy intake. Those with higher magnesium-rich diet scores were less likely to be current smokers and more likely to have poor diet quality. There was no significant difference in physical activity across the quartiles of the magnesium-rich diet score.

**Table 2:** Characteristics of the study population according to quartiles of the magnesium-rich diet score among middle-aged and older Korean adults aged ≥50 years^a^.

Magnesium-rich diet score											
			Q1 (Lowest)		Q2		Q3		Q4 (Highest)		*p* ^b^
Characteristics			Mean or *n*	SE or %	Mean or *n*	SE or %	Mean or *n*	SE or %	Mean or *n*	SE or %	
*n*			767		1,157		1,104		802		
Magnesium-rich diet score			9.38	0.02	11.53	0.02	13.42	0.02	15.78	0.04	<0.001^**^
Age (years)			61.18	0.42	62.89	0.34	62.91	0.33	64.96	0.36	<0.001^**^
Sex (%)											0.013^*^
Men			376	53.16	559	52.57	511	49.32	440	58.02	
Women			391	46.84	598	47.43	593	50.68	362	41.98	
Magnesium intake (mg/day)			251.72	4.47	302.14	4.14	349.87	4.33	419.24	5.70	<0.001^**^
BMI (kg/m^2^)			24.20	0.15	24.21	0.11	24.01	0.12	23.92	0.12	0.238
eGFR (mL/min/1.73 m^2^)			92.22	0.55	92.10	0.47	92.67	0.37	91.27	0.47	0.133
Serum vitamin D levels (ng/mL)			25.36	0.48	26.48	0.51	27.69	0.38	27.91	0.44	<0.001^**^
Physical activity^c^ (%)											0.051
Low			480	61.51	701	58.01	661	57.24	445	53.66	
High			287	38.49	456	41.99	443	42.76	357	46.34	
Marital status (%)											0.350
Married			739	96.09	1,130	97.23	1,084	97.76	779	96.88	
Unmarried			28	3.91	27	2.77	20	2.24	23	3.12	
Occupation (%)											0.117
White-collar			236	34.31	330	32.71	358	34.60	213	30.15	
Blue-collar			225	30.18	361	29.15	282	26.07	215	26.09	
Non-employed			306	35.52	464	38.14	463	39.33	373	43.75	
Education level (%)											0.048^*^
Middle school or lower			292	31.12	486	35.92	366	29.01	309	33.58	
High school			270	38.16	379	35.24	396	37.70	265	33.64	
College or higher			204	30.72	292	28.84	342	33.30	227	32.77	
Household income (%)											0.449
Lowest			176	18.96	279	20.69	217	16.94	186	20.27	
Lower-middle			197	24.05	293	23.41	285	23.98	234	27.27	
Upper-middle			168	25.08	276	25.05	283	25.96	187	24.40	
Highest			224	31.91	308	30.85	319	33.12	192	28.06	
Sleep duration (%)											0.038^*^
<6 h/day			138	18.41	194	15.91	161	14.77	106	11.97	
6–<8 h/day			410	52.86	640	56.38	642	59.18	466	59.63	
≥8 h/day			217	28.73	321	27.71	301	26.05	229	28.40	
Alcohol consumption (%)											0.325
<1 drink/day			608	77.26	916	77.43	909	80.54	661	81.24	
1–<3 drinks/day			92	13.68	98	13.77	150	13.00	128	11.87	
≥3 drinks/day			49	9.06	61	8.80	91	6.46	67	6.89	
Smoking status (%)											<0.001^**^
Non-smoker			418	50.79	638	51.55	650	56.25	438	53.40	
Past smoker			205	29.14	333	30.71	313	30.21	295	38.29	
Current smoker			144	20.06	186	17.74	141	13.54	69	8.31	
Diet quality^d^ (%)											0.044^*^
Poor			431	54.38	706	59.23	628	55.53	497	61.17	
Good			336	45.62	451	40.77	476	44.47	305	38.83	
Daily protein intake (g/day)			58.66	1.19	65.03	1.03	71.29	1.18	77.57	1.31	<0.001^**^
Daily total energy intake (kcal/day)			1642.69	26.39	1743.76	24.99	1868.85	26.07	1999.19	29.38	<0.001^**^

^a^Values are presented as means with standard errors (SEs) for continuous variables and as numbers with weighted percentages for categorical variables.

^b^The chi-square test was used to compare categorical variables across magnesium-rich diet score, and analysis of variance (*F*-test) was used to compare continuous variables across magnesium-rich diet score.

^c^High physical activity was defined as ≥150 min per week of moderate-intensity activity, ≥75 min per week of vigorous-intensity activity, or ≥150 min per week of a combination of moderate and vigorous-intensity activity, with 1 min of vigorous-intensity activity regarded as equivalent to 2 min of moderate-intensity activity.

^d^Diet quality was evaluated using a modified Diet Quality Index for Koreans and classified as poor (scores of 5–9) or good (scores of 0–4).

^*^p < 0.05; ^**^p < 0.01.

### 3.2 Magnesium intake and magnesium-rich diet score in relation to sarcopenia

The results of the multinomial logistic regression analyses on the associations of magnesium intake and the magnesium-rich diet score with sarcopenia, low muscle strength only, and low muscle mass only among middle-aged and older Korean adults are presented in Table 3 and Table 4. After full adjustment, individuals in the highest quartile of magnesium intake had 53% lower odds of sarcopenia, 54% lower odds of low muscle strength only, and 54% lower odds of low muscle mass only compared with those in the lowest quartile (*p* ≤ 0.036 for all cases). Each 100 mg/day increase in magnesium intake was associated with 26% lower odds of sarcopenia, 29% lower odds of low muscle strength only, and 21% lower odds of low muscle mass only (*p* ≤ 0.007 for all cases).

**Table 3:** Multivariable-adjusted ORs for sarcopenia, low muscle strength only, and low muscle mass only according to sex-specific quartiles of magnesium intake, per 100 mg/day, and per quartile increase in magnesium intake among middle-aged and older Korean adults aged ≥50 years.

		Q1 (Lowest)		Q2		Q3		Q4 (Highest)		Per 100 mg/day increase		Per quartile increase	
Model		OR	95% CI	OR	95% CI	OR	95% CI	OR	95% CI	OR	95% CI	OR	95% CI
No. of subjects		957		958		958		957					
Low muscle mass only													
No. of cases		219		195		190		149					
Model 1^a^		1	ref	0.80	0.62–1.02	0.76^*^	0.59–0.96	0.47^**^	0.36–0.62	0.81^**^	0.76–0.87	0.80^**^	0.74–0.87
Model 2^b^		1	ref	0.87	0.65–1.16	0.84	0.59–1.18	0.46^**^	0.29–0.74	0.79^**^	0.69–0.90	0.81^**^	0.70–0.94
Low muscle strength only													
No. of cases		52		56		47		35					
Model 1		1	ref	1.08	0.69–1.70	0.76	0.49–1.20	0.64	0.39–1.05	0.85^*^	0.74–0.96	0.85^*^	0.73–0.98
Model 2		1	ref	1.01	0.63–1.63	0.63	0.38–1.05	0.46^*^	0.23–0.91	0.71^**^	0.58–0.87	0.76^**^	0.62–0.93
Sarcopenia													
No. of cases		91		59		48		30					
Model 1		1	ref	0.54^**^	0.37–0.79	0.43^**^	0.27–0.68	0.29^**^	0.18–0.50	0.67^**^	0.57–0.79	0.66^**^	0.56–0.79
Model 2		1	ref	0.70	0.47–1.06	0.63	0.36–1.10	0.47^*^	0.23–0.95	0.74^**^	0.60–0.92	0.78^*^	0.62–0.98

OR, odds ratio; CI, confidence interval; Ref., reference.

^a^Model 1 was adjusted for age and sex.

^b^Model 2 was additionally adjusted for BMI, eGFR, physical activity, marital status, education level, occupation, household income, sleep duration, alcohol consumption, smoking status, DQI-K, daily protein intake, and daily total energy intake.

^*^p < 0.05; ^**^p < 0.01.

**Table 4:** Multivariable-adjusted ORs for sarcopenia, low muscle strength only, and low muscle mass only according to quartiles of the magnesium-rich diet score, per 1-point, and per quartile increase among middle-aged and older Korean adults aged ≥50 years.

		Q1 (Lowest)		Q2		Q3		Q4 (Highest)		Per 1-point increase		Per quartile increase	
Model		OR	95% CI	OR	95% CI	OR	95% CI	OR	95% CI	OR	95% CI	OR	95% CI
No. of subjects		767		1,157		1,104		802					
Low muscle mass only													
No. of cases		157		236		209		151					
Model 1^a^		1	ref	0.93	0.71–1.21	0.83	0.62–1.10	0.68^**^	0.51–0.90	0.94^**^	0.90–0.98	0.88^**^	0.81–0.96
Model 2^b^		1	ref	0.92	0.68–1.26	0.82	0.59–1.13	0.66^*^	0.47–0.94	0.94^*^	0.89–0.99	0.88^*^	0.78–0.98
Low muscle strength only													
No. of cases		35		69		55		31					
Model 1		1	ref	1.02	0.62–1.66	0.9	0.53–1.51	0.63	0.35–1.12	0.91^*^	0.85–0.99	0.87	0.73–1.02
Model 2		1	ref	0.98	0.60–1.62	0.83	0.49–1.41	0.52^*^	0.29–0.95	0.89^**^	0.82–0.96	0.82^*^	0.70–0.97
Sarcopenia													
No. of cases		65		64		62		37					
Model 1		1	ref	0.56^**^	0.38–0.82	0.58^**^	0.38–0.87	0.41^**^	0.26–0.65	0.87^**^	0.81–0.94	0.77^**^	0.66–0.89
Model 2		1	ref	0.59^*^	0.38–0.92	0.67	0.43–1.04	0.50^**^	0.30–0.84	0.90^*^	0.83–0.98	0.82^*^	0.70–0.97

OR, odds ratio; CI, confidence interval; Ref., reference.

^a^Model 1 was adjusted for age and sex.

^b^Model 2 was additionally adjusted for BMI, eGFR, physical activity, marital status, education level, occupation, household income, sleep duration, alcohol consumption, smoking status, DQI-K, daily protein intake, and daily total energy intake.

^*^p < 0.05; ^**^p < 0.01.

After full adjustment, individuals in the highest quartile of the magnesium-rich diet score had 50% lower odds of sarcopenia, 48% lower odds of low muscle strength only, and 34% lower odds of low muscle mass only compared with those in the lowest quartile (*p* ≤ 0.032 for all cases). The second quartile (Q2) of the magnesium-rich diet score was inversely associated with sarcopenia (OR = 0.59, *p* = 0.020), and the third quartile (Q3) showed a suggestive inverse association with sarcopenia (OR = 0.67, *p* = 0.076). Each 1-point increase in the magnesium-rich diet score was associated with 10% lower odds of sarcopenia, 11% lower odds of low muscle strength only, and 6% lower odds of low muscle mass only (*p* ≤ 0.017 for all cases).

### 3.3 Mediation analyses of the associations of magnesium intake and magnesium-rich diet score with muscle-specific parameters via high-sensitivity C-reactive protein

**Figure 2:**
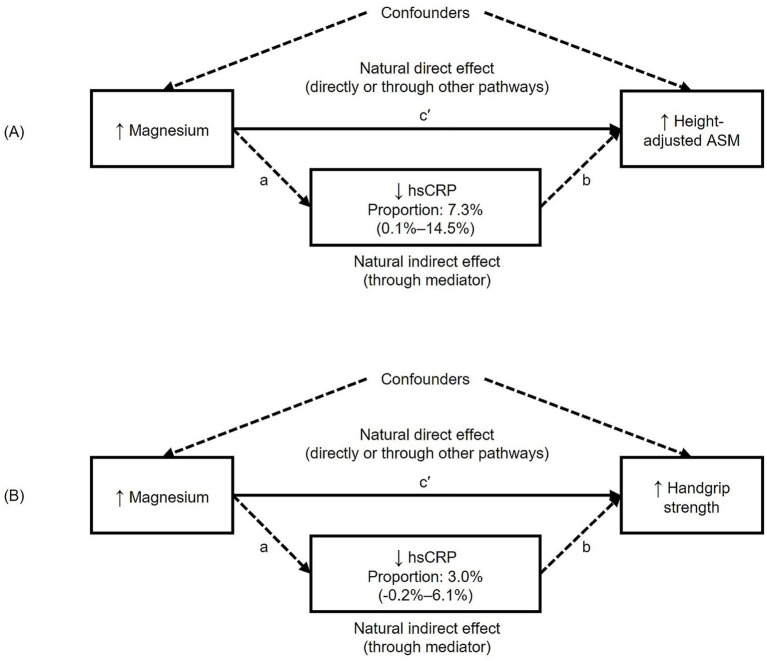
Results of mediation analyses. **(A)** Mediation analysis of the association between magnesium intake and height-adjusted ASM via hsCRP. “*a*” refers to the effect of dietary magnesium intake on log-transformed hsCRP (*β* per 100 mg/day increase in magnesium intake = −0.04, 95% CI: −0.07 to −0.004). “*b*” refers to the effect of hsCRP on height-adjusted ASM, independent of magnesium intake (*β* = −0.06, 95% CI: −0.08 to −0.05). “*c*′” refers to the effect of magnesium intake on height-adjusted ASM, operating directly or through pathways other than hsCRP (*β* per 100 mg/day increase in magnesium intake = 0.03, 95% CI: 0.01 to 0.05). Magnesium-related hsCRP partially mediated the association between magnesium intake and greater height-adjusted ASM, with a proportion mediated of 7.3%. **(B)** Mediation analysis of the association between magnesium intake and handgrip strength via hsCRP. “*a*” is the same as “*a*” described above. “*b*” refers to the effect of hsCRP on handgrip strength, independent of magnesium intake (*β* = −0.35, 95% CI: −0.52 to −0.18). “*c*′” refers to the effect of magnesium intake on handgrip strength, operating directly or through pathways other than hsCRP (*β* per 100 mg/day increase in magnesium intake = 0.44, 95% CI: 0.26 to 0.62). Given the observational nature of this study, the term “effect,” as used in the mediation analyses, should not be interpreted as a causal effect. hsCRP, high-sensitivity C-reactive protein; ASM, appendicular skeletal muscle mass; CI, confidence interval.

The results of the mediation analyses of the associations between magnesium intake and height-adjusted ASM (kg/m^2^) and handgrip strength (kg) through hsCRP are shown in Table 5 and Figure 2. The total effect size of magnesium intake (*β* per 100 mg/day increase in magnesium intake) on height-adjusted ASM was 0.03 (*p* < 0.001), of which 7.29% (*p* = 0.047) appeared to be mediated by hsCRP. The total effect size of magnesium intake (*β* per 100 mg/day increase in magnesium intake) on handgrip strength was 0.45 (*p* < 0.001), with a marginally significant proportion mediated by hsCRP of 2.96% (*p* = 0.066).

**Table 5:** Mediation analyses of the associations between magnesium intake and muscle-specific parameters via hsCRP among middle-aged and older Korean adults aged ≥50 years.

β per 100 mg/day increase in magnesium intake and the corresponding 95% CI^a^								
	Total effect		Direct effect		Indirect effect		Percentage mediated and 95% CI^b^	
Height-adjusted ASM (kg/m^2^)	0.0324^**^	0.0167–0.0480	0.0300^**^	0.0145–0.0455	0.0024^*^	0.0002–0.0045	7.2932^*^	0.0992–14.4873
Handgrip strength (kg)	0.4532^**^	0.2732–0.6333	0.4398^**^	0.2601–0.6195	0.0134	−0.0002–0.0270	2.9606	−0.1992–6.1204

hsCRP, high-sensitivity C-reactive protein; CI, confidence interval; ASM, appendicular skeletal muscle mass.

^a^Models were adjusted for age, sex, BMI, eGFR, physical activity, marital status, education level, occupation, household income, sleep duration, alcohol consumption, smoking status, DQI-K, daily protein intake, and daily total energy intake.

^b^Proportion of the indirect effect through hsCRP relative to the total effect of magnesium intake on each muscle-specific parameter.

^*^p < 0.05; ^**^p < 0.01.

**Figure 3:**
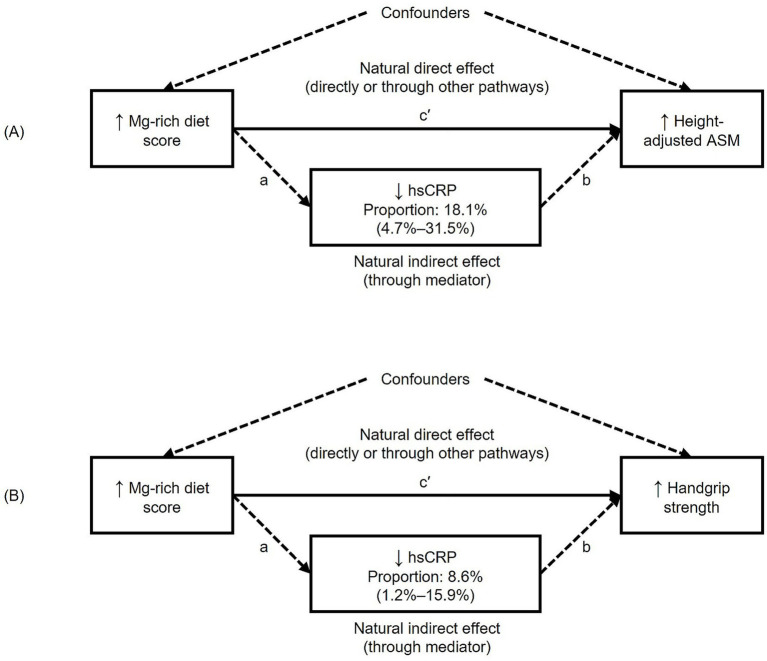
Results of mediation analyses. **(A)** Mediation analysis of the association between the magnesium-rich diet score and height-adjusted ASM via hsCRP. “a” refers to the effect of the magnesium-rich diet score on log-transformed hsCRP (β per 1-point increase in the magnesium-rich diet score = −0.03, 95% CI: −0.05 to −0.02). “b” refers to the effect of hsCRP on height-adjusted ASM, independent of the magnesium-rich diet score (*β* = −0.06, 95% CI: −0.08 to −0.05). “c′” refers to the effect of the magnesium-rich diet score on height-adjusted ASM, operating directly or through pathways other than hsCRP (*β* per 1-point increase in the magnesium-rich diet score = 0.01, 95% CI: 0.002 to 0.02). Magnesium-related hsCRP partially mediated the association between the magnesium-rich diet score and greater height-adjusted ASM, with a proportion mediated of 18.09%. **(B)** Mediation analysis of the association between the magnesium-rich diet score and handgrip strength via hsCRP. “a” is the same as Path A described above. “b” refers to the effect of hsCRP on handgrip strength, independent of the magnesium-rich diet score (*β* = −0.35, 95% CI: −0.52 to −0.18). “c′” refers to the effect of the magnesium-rich diet score on handgrip strength, operating directly or through pathways other than hsCRP (*β* per 1-point increase in the magnesium-rich diet score = 0.12, 95% CI: 0.04 to 0.19). Magnesium-related hsCRP partially mediated the association between the magnesium-rich diet score and greater handgrip strength, with a proportion mediated of 8.57%. Given the observational nature of this study, the term “effect,” as used in the mediation analyses, should not be interpreted as a causal effect. hsCRP, high-sensitivity C-reactive protein; ASM, appendicular skeletal muscle mass; CI, confidence interval.

The results of the mediation analyses of the associations between the magnesium-rich diet score and height-adjusted ASM (kg/m^2^) and handgrip strength (kg) through hsCRP are shown in Table 6 and Figure 3. The total effect size of the magnesium-rich diet score (*β* per 1-point increase) on height-adjusted ASM was 0.01 (*p* = 0.001), of which 18.09% (*p* = 0.008) appeared to be mediated by hsCRP. The total effect size of the magnesium-rich diet score (*β* per 1-point increase) on handgrip strength was 0.13 (*p* < 0.001), with a proportion mediated of 8.57% (*p* = 0.023).

**Table 6:** Mediation analyses of the associations between the magnesium-rich diet score and muscle-specific parameters via hsCRP among middle-aged and older Korean adults aged ≥50 years.

β per 1-point increase in the magnesium-rich diet score and the corresponding 95% CI^a^								
	Total effect		Direct effect		Indirect effect		Percentage mediated and 95% CI^b^	
Height-adjusted ASM (kg/m^2^)	0.0107^**^	0.0042–0.0172	0.0088^**^	0.0024–0.0152	0.0019^**^	0.0010–0.0029	18.0869^**^	4.7068–31.4669
Handgrip strength (kg)	0.1280^**^	0.0536–0.2024	0.1170^**^	0.0426–0.1914	0.0110^**^	0.0037–0.0182	8.5656^*^	1.2052–15.9261

hsCRP, high-sensitivity C-reactive protein; CI, confidence interval; ASM, appendicular skeletal muscle mass.

^a^Models were adjusted for age, sex, BMI, eGFR, physical activity, marital status, education level, occupation, household income, sleep duration, alcohol consumption, smoking status, DQI-K, daily protein intake, and daily total energy intake.

^b^Proportion of the indirect effect through hsCRP relative to the total effect of magnesium-rich diet score on each muscle-specific parameter.

^*^p < 0.05; ^**^p < 0.01.

Additionally, magnesium intake and the magnesium-rich diet score were inversely associated with hsCRP (*β* per 100 mg/day increase in magnesium intake = −0.04, *p* = 0.028; *β* per 1-point increase in the magnesium-rich diet score = −0.03, *p* < 0.001). hsCRP was inversely associated with height-adjusted ASM (*β* = −0.06, *p* < 0.001) and handgrip strength (*β* = −0.35, *p* < 0.001), independent of magnesium intake.

### 3.4 Protein intake and serum vitamin D in relation to sarcopenia

The results of the multinomial logistic regression analyses on the associations of protein intake and serum vitamin D levels with sarcopenia, low muscle strength only, and low muscle mass only are presented in the Supplementary Table S1 and Supplementary Table S2. After further adjustment for magnesium intake, we found that higher protein intake and higher serum vitamin D levels were marginally associated with lower odds of sarcopenia overall. The results of the multinomial logistic regression analyses on the associations of magnesium intake with sarcopenia, low muscle strength only, and low muscle mass only, with additional adjustment for serum vitamin D levels, are also presented in the Supplementary Table S3. Higher magnesium intake was associated with lower odds of sarcopenia (OR per 100 mg/day increase in magnesium intake = 0.75, *p* = 0.009), independent of serum vitamin D levels.

## 4 Discussion

This study investigated the associations of magnesium intake and a magnesium-rich diet score with sarcopenia in 3,830 middle-aged and older adults using data from KNHANES, a nationally representative study of the Korean population. We found that the highest quartile of magnesium intake (≥351 mg/day for women and ≥442 mg/day for men) was inversely associated with sarcopenia, low muscle strength only, and low muscle mass only compared with the lowest quartile. Each 100 mg/day increase in magnesium intake was associated with 26% lower odds of sarcopenia, and the inverse association remained robust after further adjustment for serum vitamin D levels. The highest quartile of the magnesium-rich diet score was also inversely associated with sarcopenia, and each 1-point increase was associated with 10% lower odds of sarcopenia. hsCRP appeared to partially mediate the association between magnesium intake and greater height-adjusted ASM, with a proportion mediated of 7.3%. hsCRP also appeared to partially mediate the associations of the magnesium-rich diet score with greater height-adjusted ASM and handgrip strength, with proportions mediated of 18.1 and 8.6%, respectively.

Consistent with our observed inverse association of magnesium intake and the magnesium-rich diet score with sarcopenia, a cross-sectional study reported a dose-dependent relationship between oral magnesium intake and sarcopenia, as defined by the EWGSOP2 diagnostic criteria, among U.S. older adults aged 60–85 years (17). Previous studies have also examined magnesium intake or circulating magnesium levels in relation to various muscle-specific parameters; however, the findings remain inconsistent. For example, a large-scale cross-sectional study using UK Biobank data reported a positive association between magnesium intake and grip strength in individuals aged ≥60 years (19). Another cross-sectional study using TwinsUK data observed a positive association between dietary magnesium intake and skeletal muscle mass and power in adult women, and found no significant association with grip strength (18). On the other hand, a randomized controlled trial (RCT) involving 74 overweight Iranian women aged 40–55 years reported that 250 mg/day of magnesium oxide, equivalent to 150 mg/day of elemental magnesium, for 8 weeks did not result in significant improvements in muscle strength or function compared with placebo (20). Another cross-sectional study of 71 Indonesian older adults aged ≥60 years reported no significant association between magnesium intake and handgrip strength (21). These null findings may be partly attributable to smaller sample sizes and limited exposure contrast. For example, in the present study, the difference in magnesium intake between the lowest and highest quartiles was approximately 270 mg/day for women and 320 mg/day for men, which was comparable to the exposure contrasts observed in previous studies reporting favorable associations between magnesium intake and muscle health (18, 44). By contrast, the RCT provided magnesium oxide equivalent to 150 mg/day of elemental magnesium, and the Indonesian cross-sectional study dichotomized magnesium intake using sex-specific adequacy cutoffs, which may have limited the exposure contrast.

The mechanisms underlying the inverse association between magnesium intake and sarcopenia can be explained by magnesium’s various roles in muscle health, including protein synthesis, energy metabolism, and anti-inflammatory and antioxidant activities. First, aging leads to anabolic resistance, in which the capacity for protein synthesis is reduced. Anabolic resistance can persist even in the presence of typical anabolic stimuli, such as feeding and exercise (15). The mTOR plays an important role in promoting protein synthesis while simultaneously inhibiting proteolysis. Magnesium activates mTOR signaling and supports RNA synthesis and ribosomal stabilization, thereby supporting muscle protein synthesis (15, 45). Second, most intracellular ATP exists as Mg–ATP complexes, which represent the biologically active form of ATP (15). The Mg–ATP complex is a key component of the sliding filament mechanism underlying myofibrillar contraction and relaxation in striated muscle. Furthermore, magnesium deficiency can impair mitochondrial function by stimulating the uncoupling of oxidative phosphorylation, resulting in electron loss from the electron transport chain and increased reactive oxygen species generation, thereby promoting oxidative stress (15). Third, oxidative stress and inflammation can disturb the balance between protein synthesis and degradation and impair mitochondrial function. Magnesium deficiency is characterized by compromised antioxidant defense and elevated levels of oxidative stress markers, such as lipid, protein, and DNA oxidative modification products (46). Moreover, cumulative evidence indicates that NF-κB is a key signaling pathway related to the loss of skeletal muscle mass (15). Meta-analyses and large-scale studies have reported that dietary magnesium intake or magnesium supplementation is inversely associated with CRP levels (23, 24, 47). A recent umbrella review suggested that inflammatory biomarkers, including IL-6, CRP, and TNF-*α*, constitute a predominant class of biomarkers in sarcopenia research and are generally elevated in patients with sarcopenia (27). Relatedly, a population-based study involving U. S. adults reported that a higher oxidative balance score, indicating a more favorable balance between pro-oxidants and antioxidants related to dietary and lifestyle factors, was associated with lower odds of sarcopenia and osteosarcopenia (48). Recent studies have also suggested that hsCRP may partially mediate the associations of dietary magnesium intake or serum trace elements with chronic disease outcomes (49, 50). In addition, the mechanisms underlying the inverse association between the magnesium-rich diet score and sarcopenia may be explained not only by magnesium but also by various anti-inflammatory and antioxidant components in magnesium-rich plant foods, such as isoflavones in soy foods, chlorogenic acid and other polyphenols in coffee, vitamin C and other phytochemicals in vegetables, and dietary fiber and polyphenols in whole grains (51–54).

Using mediation analysis, we found modest potential mediating roles of hsCRP in the beneficial associations of magnesium intake and the magnesium-rich diet score with muscle-specific parameters. These modest mediation proportions may reflect magnesium’s diverse roles in muscle health beyond its anti-inflammatory activities, such as supporting muscle protein synthesis and energy metabolism (15). Furthermore, hsCRP alone may not fully reflect inflammatory status, and data on other inflammatory biomarkers or sarcopenia-related proteomic signatures were limited in this study. hsCRP is a downstream marker of chronic systemic inflammation and is produced in response to increased levels of pro-inflammatory cytokines, including IL-6 and IL-1β (22, 55). A longitudinal multicohort study conducted in South Korea identified and validated plasma protein signatures associated with sarcopenia progression, with pathway enrichment analyses highlighting acute-phase response signaling and complement cascade activation as key mechanisms (56). A cross-sectional study involving 2,750 U.K. women reported that the inverse association between hsCRP levels and skeletal muscle mass was attenuated by 6.5% after additional adjustment for magnesium intake (18), which aligns with our mediation results. However, this previous study did not perform a mediation analysis and was restricted to women (18). In our study, hsCRP appeared to mediate a greater proportion of the associations between the magnesium-rich diet score and muscle-specific parameters than between magnesium intake and these parameters. These findings may reflect complementary effects of nutrients and foods within the magnesium-rich dietary pattern (29, 30), as well as the contribution of other anti-inflammatory and antioxidant components (51–54).

In this study, the inverse association between magnesium intake and sarcopenia was stronger, while the inverse associations of protein intake and serum vitamin D levels with sarcopenia were weaker, which is partially consistent with findings from previous studies (18, 19). In a large-scale cross-sectional study using the UK Biobank, magnesium exhibited the largest standardized beta coefficient for grip strength among both macronutrients and micronutrients (19). A previous cross-sectional study conducted in the U. K. compared the magnitude of the associations of magnesium and protein intake with muscle mass and reported a 3 to 7-fold stronger association for magnesium intake than for protein intake (18). Magnesium influences both transcriptional and translational processes in protein synthesis in skeletal muscle (15). Furthermore, magnesium assists in vitamin D activation and appears to be required as a cofactor for enzymes that metabolize vitamin D (57). Studies comparing the magnitude of associations of magnesium, protein, and vitamin D with sarcopenia remain limited and warrant further investigation. A meta-analysis of RCTs reported that whey protein, leucine, and vitamin D supplementation may increase appendicular muscle mass in patients with sarcopenia; however, muscle strength and physical function improved only when the supplementation was combined with physical exercise (58). A recent RCT among older Malaysian adults reported that leucine-rich, high-protein supplementation did not significantly alter body composition or muscle function, although it may improve gene expression (59). A previous study utilizing the TwinsUK cohort reported that high protein intake was associated with sarcopenia in healthy older twins, possibly because individuals with sarcopenia may have deliberately consumed more protein to mitigate muscle loss (60). On the other hand, a recent cross-sectional study conducted in China suggested that high intake of dietary branched-chain amino acids, including leucine, isoleucine, and valine, was associated with lower odds of sarcopenia in older adults (61).

The difference in median magnesium intake between Q1 and Q4 was approximately 270 mg/day for women and 320 mg/day for men. A RCT involving 83 healthy middle-aged Iranian women with vitamin D deficiency reported that co-supplementation of vitamin D (50,000 IU/week) and magnesium (250 mg/day) for 8 weeks improved muscle strength, muscle function, and possibly inflammation compared with placebo. Although the intervention involved a combination of vitamin D and magnesium, the magnesium dose of 250 mg/day is comparable to the 270 mg/day difference observed in women in our study (44). The difference in median magnesium intake between the extreme quartiles can be achieved through a plant-based diet that includes magnesium-rich foods. Dietary sources of magnesium include dark green leafy vegetables [e.g., boiled spinach, 1/2 cup (90 g), 78 mg], unrefined grains [e.g., shredded wheat cereal, two biscuits (42 g), 72 mg], nuts, and legumes [e.g., dry-roasted cashews, 1 ounce, 74 mg; soymilk, 1 cup (244 g), 54 mg] (12). According to the KDRIs, the tolerable upper intake level for magnesium has not been established for food sources but has been set at 350 mg/day for non-food sources (38). Serum magnesium levels are tightly regulated within the range of 0.75–0.95 mmol/L by the balance of intestinal Mg^2+^ absorption and renal Mg^2+^ excretion (62). If renal function is not impaired, higher dietary magnesium intake is unlikely to exert adverse effects (63); however, further research is warranted. In the present study, individuals with kidney disease were excluded, and eGFR was adjusted for in the statistical models.

To the best of our knowledge, this is the first study to examine the associations of magnesium intake and a magnesium-rich diet score with sarcopenia, while assessing the potential mediating role of hsCRP in these associations. We found robust inverse associations of both magnesium intake and the magnesium-rich diet score with sarcopenia. Because we used nationally representative data from the Korean population, our findings can be generalized to middle-aged and older Koreans. We also applied the updated AWGS diagnostic criteria for sarcopenia, which were newly expanded to include middle-aged adults. Nevertheless, this study has several limitations. First, because of the cross-sectional design, the temporal sequence cannot be inferred, and causal inferences are limited. Second, a single 24-h dietary recall may not fully represent usual magnesium intake and may have introduced exposure misclassification. Such misclassification could have attenuated the observed associations, as it was likely non-differential and might have made the exposure groups more similar (64). Nevertheless, a controlled-feeding study including older Korean women reported that a single interviewer-administered 24-h dietary recall can be a reliable tool for assessing food and nutrient intake (65). Third, although excluding individuals with related chronic diseases, such as cancer, renal disease, and cardiovascular disease, may have reduced the possibility of reverse causation, this exclusion may limit the generalizability of our findings to individuals with these diseases, in whom dietary intake, inflammatory status, and muscle metabolism may differ. Fourth, although we adjusted for multiple confounders, including diet quality assessed using the DQI-K, protein intake, and total energy intake, residual confounding by unmeasured variables, such as supplement intake and medication use, may persist. Fifth, because no significant interactions by sex or vitamin D status were observed, further stratified analyses by these variables were not performed. Future prospective studies with larger sample sizes are warranted to test potential effect modification by these variables. Sixth, because data on magnesium intake from supplements remain limited in KNHANES, possibly because the prevalence of magnesium supplement use is relatively low (31), our findings reflect dietary magnesium intake only, not total magnesium intake. Seventh, although the complex survey design of KNHANES was accounted for in the descriptive analyses and logistic regression models, it was not incorporated into the mediation analyses because design-based mediation analysis was not available within the statistical procedure used in this study. Therefore, the mediation findings should be interpreted with caution. Finally, because muscle-specific parameters were measured only once, random measurement error would likely attenuate the observed associations toward the null.

In conclusion, higher dietary magnesium intake and magnesium-rich diet score were associated with lower odds of sarcopenia in middle-aged and older Korean adults, and lower chronic inflammation appears to partially mediate these associations. Given that the age-related loss of skeletal muscle mass and function occurs in all individuals, potential effects of adequate magnesium intake may be large on a population scale. While adequate protein intake is emphasized in dietary guidelines for maintaining muscle health, our findings suggest that consuming magnesium-rich plant foods, such as whole grains, nuts and legumes, green leafy vegetables and seaweeds, soymilk, and coffee, as part of a balanced diet may also help support muscle health in middle-aged and older adults. Well-designed prospective studies are warranted to clarify the associations between magnesium intake and incident sarcopenia and longitudinal changes in muscle-specific parameters, and to explore the mediating roles of various inflammatory markers and proteomic signatures.

## Data Availability

Publicly available datasets were analyzed in this study. This data can be found at: https://knhanes.kdca.go.kr/knhanes/eng/main.do.
