# Supplementary material for: Associations of magnesium intake and a magnesium-rich diet score with sarcopenia in middle-aged and older Korean adults: potential mediation by C-reactive protein
Source: Front Nutr. 2026 Aug 3;13:1854192. doi: 10.3389/fnut.2026.1854192 (PMC13477984; doi:10.3389/fnut.2026.1854192)
Supplement: Supplementary file 1 [file Table_1.docx]

Supplementary Material

**Supplementary Table 1**. Multivariable-adjusted ORs for sarcopenia, low muscle strength only, and low muscle mass only according to sex-specific quartiles of protein intake, per 10 g/day, and per quartile increase in protein intake among middle-aged and older Korean adults aged ≥50 years

|  |  | Q1 (Lowest) | |  | Q2 | |  | Q3 | |  | Q4 (Highest) | |  | Per 10 g/day Increase | |  | Per Quartile Increase | |
| --- | --- | --- | --- | --- | --- | --- | --- | --- | --- | --- | --- | --- | --- | --- | --- | --- | --- | --- |
|  |  | OR | 95% CI |  | OR | 95% CI |  | OR | 95% CI |  | OR | 95% CI |  | OR | 95% CI |  | OR | 95% CI |
| No. of subjects | | 957 |  |  | 958 |  |  | 958 |  |  | 957 |  |  |  |  |  |  |  |
| Low muscle mass only | | |  |  |  |  |  |  |  |  |  |  |  |  |  |  |  |  |
| No. of cases | | 214 |  |  | 204 |  |  | 192 |  |  | 143 |  |  |  |  |  |  |  |
| Model 1^a^ |  | 1 | ref |  | 0.86 | 0.68–1.10 |  | 0.81 | 0.65–1.03 |  | 0.54^**^ | 0.42–0.70 |  | 0.92^**^ | 0.89–0.95 |  | 0.83^**^ | 0.77–0.90 |
| Model 2^b^ |  | 1 | ref |  | 0.99 | 0.72–1.35 |  | 1.07 | 0.72–1.59 |  | 0.83 | 0.47–1.46 |  | 1.02 | 0.94–1.11 |  | 0.97 | 0.81–1.16 |
| Low muscle strength only | | |  |  |  |  |  |  |  |  |  |  |  |  |  |  |  |  |
| No. of cases | | 53 |  |  | 43 |  |  | 51 |  |  | 43 |  |  |  |  |  |  |  |
| Model 1 |  | 1 | ref |  | 0.67 | 0.41–1.10 |  | 0.73 | 0.47–1.14 |  | 0.79^**^ | 0.50–1.25 |  | 0.98 | 0.92–1.04 |  | 0.94 | 0.80–1.10 |
| Model 2 |  | 1 | ref |  | 0.85 | 0.49–1.46 |  | 1.16 | 0.64–2.13 |  | 1.71 | 0.74–3.93 |  | 1.12 | 0.99–1.26 |  | 1.19 | 0.90–1.57 |
| Sarcopenia | |  |  |  |  |  |  |  |  |  |  |  |  |  |  |  |  |  |
| No. of cases | | 91 |  |  | 62 |  |  | 50 |  |  | 25 |  |  |  |  |  |  |  |
| Model 1 |  | 1 | ref |  | 0.60^*^ | 0.39–0.90 |  | 0.53^**^ | 0.34–0.83 |  | 0.25^**^ | 0.14–0.43 |  | 0.83^**^ | 0.77–0.90 |  | 0.66^**^ | 0.56–0.78 |
| Model 2 |  | 1 | ref |  | 0.75 | 0.45–1.25 |  | 0.77 | 0.39–1.52 |  | 0.44 | 0.18–1.09 |  | 0.94 | 0.81–1.09 |  | 0.81 | 0.61–1.09 |

OR, odds ratio; CI, confidence interval; Ref., reference.
^a^ Model 1 was adjusted for age and sex.
^b^ Model 2 was additionally adjusted for BMI, eGFR, physical activity, marital status, education level, occupation, household income, sleep duration, alcohol consumption, smoking status, DQI-K, daily total energy intake, and daily magnesium intake.
^*^ *P* < 0.05; ^**^ *P* < 0.01.

**Supplementary Table 2**. Multivariable-adjusted ORs for sarcopenia, low muscle strength only, and low muscle mass only according to serum vitamin D quartiles, per 5 ng/mL, and per quartile increase in vitamin D levels among middle-aged and older Korean adults aged ≥50 years

|  |  | Q1 (Lowest) | |  | Q2 | |  | Q3 | |  | Q4 (Highest) | |  | Per 5 ng/mL Increase | |  | Per Quartile Increase | |
| --- | --- | --- | --- | --- | --- | --- | --- | --- | --- | --- | --- | --- | --- | --- | --- | --- | --- | --- |
|  |  | OR | 95% CI |  | OR | 95% CI |  | OR | 95% CI |  | OR | 95% CI |  | OR | 95% CI |  | OR | 95% CI |
| No. of subjects | | 957 |  |  | 958 |  |  | 958 |  |  | 957 |  |  |  |  |  |  |  |
| Low muscle mass only | | |  |  |  |  |  |  |  |  |  |  |  |  |  |  |  |  |
| No. of cases | | 217 |  |  | 200 |  |  | 199 |  |  | 137 |  |  |  |  |  |  |  |
| Model 1^a^ |  | 1 | ref |  | 0.76^*^ | 0.59–0.98 |  | 1.01 | 0.78–1.29 |  | 0.99 | 0.77–1.28 |  | 1.03 | 0.99–1.08 |  | 1.02 | 0.94–1.11 |
| Model 2^b^ |  | 1 | ref |  | 0.83 | 0.61–1.12 |  | 0.97 | 0.72–1.29 |  | 0.88 | 0.64–1.21 |  | 1.00 | 0.96–1.05 |  | 0.98 | 0.88–1.08 |
| Low muscle strength only | | |  |  |  |  |  |  |  |  |  |  |  |  |  |  |  |  |
| No. of cases | | 56 |  |  | 48 |  |  | 49 |  |  | 37 |  |  |  |  |  |  |  |
| Model 1 |  | 1 | ref |  | 0.84 | 0.51–1.37 |  | 0.95 | 0.58–1.54 |  | 0.84 | 0.52–1.36 |  | 1.00 | 0.92–1.08 |  | 0.96 | 0.82–1.12 |
| Model 2 |  | 1 | ref |  | 0.84 | 0.51–1.39 |  | 0.98 | 0.59–1.62 |  | 0.86 | 0.52–1.45 |  | 1.00 | 0.93–1.09 |  | 0.97 | 0.82–1.15 |
| Sarcopenia | |  |  |  |  |  |  |  |  |  |  |  |  |  |  |  |  |  |
| No. of cases | | 85 |  |  | 75 |  |  | 39 |  |  | 29 |  |  |  |  |  |  |  |
| Model 1 |  | 1 | ref |  | 0.46^**^ | 0.3–0.71 |  | 0.70 | 0.46–1.08 |  | 0.68 | 0.45–1.02 |  | 0.93 | 0.85–1.01 |  | 0.90 | 0.77–1.04 |
| Model 2 |  | 1 | ref |  | 0.54^*^ | 0.34–0.88 |  | 0.73 | 0.45–1.16 |  | 0.66 | 0.42–1.03 |  | 0.93 | 0.86–1.01 |  | 0.89 | 0.76–1.04 |

OR, odds ratio; CI, confidence interval; Ref., reference.
^a^ Model 1 was adjusted for age and sex.
^b^ Model 2 was additionally adjusted for BMI, eGFR, physical activity, marital status, education level, occupation, household income, sleep duration, alcohol consumption, smoking status, DQI-K, daily protein intake, daily total energy intake, and daily magnesium intake.
^*^ *P* < 0.05; ^**^ *P* < 0.01.

**Supplementary Table 3**. Multivariable-adjusted ORs for sarcopenia, low muscle strength only, and low muscle mass only according to sex-specific quartiles of magnesium intake, per 100 mg/day, and per quartile increase in magnesium intake, with further adjustment for serum vitamin D levels, among middle-aged and older Korean adults aged ≥50 years

|  |  | Q1 (Lowest) | |  | Q2 | |  | Q3 | |  | Q4 (Highest) | |  | Per 100 mg/day Increase | |  | Per Quartile Increase | |
| --- | --- | --- | --- | --- | --- | --- | --- | --- | --- | --- | --- | --- | --- | --- | --- | --- | --- | --- |
|  |  | OR | 95% CI |  | OR | 95% CI |  | OR | 95% CI |  | OR | 95% CI |  | OR | 95% CI |  | OR | 95% CI |
| No. of subjects | | 957 |  |  | 958 |  |  | 958 |  |  | 957 |  |  |  |  |  |  |  |
| Low muscle mass only | | |  |  |  |  |  |  |  |  |  |  |  |  |  |  |  |  |
| No. of cases | | 219 |  |  | 195 |  |  | 190 |  |  | 149 |  |  |  |  |  |  |  |
| Model 1^a^ |  | 1 | ref |  | 0.80 | 0.62–1.02 |  | 0.76^*^ | 0.59–0.96 |  | 0.47^**^ | 0.36–0.62 |  | 0.81^**^ | 0.76–0.87 |  | 0.80^**^ | 0.74–0.87 |
| Model 2^b^ |  | 1 | ref |  | 0.87 | 0.65–1.17 |  | 0.83 | 0.58–1.18 |  | 0.45^*^ | 0.27–0.73 |  | 0.78^**^ | 0.68–0.90 |  | 0.80^**^ | 0.69–0.93 |
| Low muscle strength only | | |  |  |  |  |  |  |  |  |  |  |  |  |  |  |  |  |
| No. of cases | | 52 |  |  | 56 |  |  | 47 |  |  | 35 |  |  |  |  |  |  |  |
| Model 1 |  | 1 | ref |  | 1.08 | 0.69–1.70 |  | 0.76 | 0.49–1.20 |  | 0.64 | 0.39–1.05 |  | 0.85^*^ | 0.74–0.96 |  | 0.85^*^ | 0.73–0.98 |
| Model 2 |  | 1 | ref |  | 1.02 | 0.63–1.66 |  | 0.64 | 0.38–1.07 |  | 0.45^*^ | 0.22–0.92 |  | 0.71^**^ | 0.57–0.88 |  | 0.76^**^ | 0.61–0.93 |
| Sarcopenia | |  |  |  |  |  |  |  |  |  |  |  |  |  |  |  |  |  |
| No. of cases | | 91 |  |  | 59 |  |  | 48 |  |  | 30 |  |  |  |  |  |  |  |
| Model 1 |  | 1 | ref |  | 0.54^**^ | 0.37–0.79 |  | 0.43^**^ | 0.27–0.68 |  | 0.29^**^ | 0.18–0.50 |  | 0.67^**^ | 0.57–0.79 |  | 0.66^**^ | 0.56–0.79 |
| Model 2 |  | 1 | ref |  | 0.73 | 0.49–1.10 |  | 0.64 | 0.37–1.13 |  | 0.49^*^ | 0.24–0.99 |  | 0.75^**^ | 0.61–0.93 |  | 0.79^*^ | 0.63–0.99 |

OR, odds ratio; CI, confidence interval; Ref., reference.
^a^ Model 1 was adjusted for age and sex.
^b^ Model 2 was additionally adjusted for BMI, eGFR, physical activity, marital status, education level, occupation, household income, sleep duration, alcohol consumption, smoking status, DQI-K, daily protein intake, daily total energy intake, and serum vitamin D levels.
^*^ *P* < 0.05; ^**^ *P* < 0.01.
